# Framing Environmental Health Decision-Making: The Struggle over Cumulative Impacts Policy

**DOI:** 10.3390/ijerph18083947

**Published:** 2021-04-09

**Authors:** Devon C. Payne-Sturges, Thurka Sangaramoorthy, Helen Mittmann

**Affiliations:** 1 Maryland Institute for Applied Environmental Health, School of Public Health, University of Maryland, 2234 L SPH, 255 Valley Drive, College Park, MD 20742, USA; 2 Department of Anthropology, University of Maryland, 1111 Woods Hall, 4302 Chapel Lane, College Park, MD 20742, USA; tsangara@umd.edu; 3 Department of Health Policy and Management, Milken Institute School of Public Health, George Washington University, 950 New Hampshire Avenue, NW, Washington, DC 20052, USA; hmittmann@email.gwu.edu

**Keywords:** US, cumulative risk, framing theory, environmental justice, public policy, health disparities, ethnographic research

## Abstract

Little progress has been made to advance U.S. federal policy responses to growing scientific findings about cumulative environmental health impacts and risks, which also show that many low income and racial and ethnic minority populations bear a disproportionate share of multiple environmental burdens. Recent scholarship points to a “standard narrative” by which policy makers rationalize their slow efforts on environmental justice because of perceived lack of data and analytical tools. Using a social constructivist approach, ethnographic research methods, and content analysis, we examined the social context of policy challenges related to cumulative risks and impacts in the state of Maryland between 2014 and 2016. We identified three frames about cumulative impacts as a health issue through which conflicts over such policy reforms materialize and are sustained: (a) perceptions of evidence, (b) interpretations of social justice, and (c) expectations of authoritative bodies. Our findings illustrate that policy impasse over cumulative impacts is highly dependent on how policy-relevant actors come to frame issues around legislating cumulative impacts, rather than the “standard narrative” of external constraints. Frame analysis may provide us with more robust understandings of policy processes to address cumulative risks and impacts and the social forces that create health policy change.

## 1. Introduction

Americans’ daily environmental exposures to multiple chemical compounds are well documented [1]. Moreover, evidence shows that many low income and racial and ethnic minority populations bear a disproportionate share of these exposures [2,3]. Scholars widely recognize that chemical toxicants in the environment interact in myriad predicted and unforeseen ways such that adverse health effects of combined exposures to toxicants may increase multiplicatively compared to the additive effects of individual agents [4,5]. Further, increasing evidence demonstrates that non-chemical stressors, including psychosocial factors, heighten people’s vulnerability to the adverse health effects of combined exposures [3,6,7,8,9]. Community advocates, stakeholders, and independent advisory bodies have urged local and federal environmental agencies to improve risk assessment and risk management practices to better account for multiple stressors that cumulatively impact community and population health [5,10]. While significant research investments have been made to develop such assessment methods [11,12,13,14], coherent strategies to mitigate cumulative effects of multiple environmental contaminant and stressor exposures on human health, especially in the policy arena, continue to lag.

To date, most scholarship on cumulative environmental health risks and impacts has focused on addressing the lack of information on cumulative health effects, the development of more precise technical tools, analytical procedures and risk assessment frameworks, and legal authorities [12,13,15,16,17,18,19,20,21,22,23,24,25]. This body of work has largely centered on exogenous constraints as causes for the slow progress of policy action on addressing cumulative risks and impacts. Some states have attempted to overcome cumulative risk and impacts policy challenges by employing strategies that seemingly address such barriers vis-à-vis surveillance measures [26,27], local zoning ordinances [28] and regulatory mechanisms [29,30,31,32].

The State of Maryland, likewise, has pursued remedies related to cumulative impacts. During each legislative session of the Maryland General Assembly from 2014–2016, a bill was introduced on cumulative impacts. In 2014, House Bill (HB)1210/ Senate Bill (SB)706: Permit Determinations Cumulative Impact Assessments was proposed, requiring industrial applicants to conduct cumulative impact assessments of their operations on the environment and on human populations before being granted permits. While this bill was successfully voted upon by the Maryland State Senate with revisions, it failed to pass out from the House Environmental Matters Committee. In response to the political impasse, subsequent bills were introduced during the next two legislative sessions which shifted the focus from cumulative impacts to a broader emphasis on environmental justice. These bills also did not pass, and since then, there have not been any cumulative impacts legislative proposals in Maryland.

We employed ethnographic methodology to study the social context of policy challenges related to cumulative risks and impacts. Ethnography is an approach and a qualitative research method historically used by cultural anthropologists to provide insight into the processes and meanings that sustain and motivate social groups [33]. In this paper, we report on qualitative and ethnographic data collected through in-depth interviews, participant observation, and content analysis of state-level legislative sessions. We examined how policy-relevant actors (e.g., community residents, advocates, law makers, governmental agency staff, business leaders and lobbyists) frame cumulative risks and impacts to better understand the complex social factors that hinder efforts to enact cumulative impacts policy in Maryland. We utilized a social constructionist approach [34] to illustrate how policy-relevant actors’ framing of cumulative impacts precludes the development of policy responses in Maryland despite growing evidence of cumulative adverse health effects of multiple chemical and non-chemical exposures.

### 1.1. Cumulative Risk Policy Context

One of the most enduring controversies related to environmental decision making is the need to evaluate the combined (cumulative) risk to human health from concurrent exposures to multiple environmental stressors. Below, we provide a brief overview of cumulative risk policy at the federal level to contextualize cumulative impacts legislation in Maryland.

Early development of risk assessment methodology closely followed the U.S. Environmental Protection Agency (EPA)’s strategy for pollution control, which was directed toward assessing and controlling individual chemicals [35,36]. Risk assessment played an important role in many federal regulatory decisions, bolstered by the 1980 Supreme Court decision, Industrial Union Department, ALF-CIO v. American Petroleum Institute (e.g., “Benzene Decision”) which sent a strong signal that quantitative risk assessment was necessary to justify regulatory intervention. In 1983, the National Research Council (NRC) published its landmark report, the “Red Book,” which synthesized relevant concepts and recommended standardization of risk assessment practice, leading to more full scale adoption of risk assessment for environmental decision making [37]. EPA followed these NRC recommendations, publishing a series of risk assessment guidelines. The emphasis, however, continued to be on the assessment of human health risks of *single* chemical exposures.

The limitations of chemical-by-chemical risk assessment to address real world exposures soon became evident [24]. The need to determine cleanup standards for thousands of hazardous waste sites contaminated with multiple chemicals required that risk prioritization account for exposures to chemical mixtures [38]. Community-based environmental justice actions throughout the 1980s and 1990s on cumulative effects of pollution among racial and ethnic minority communities further emphasized the need for cumulative human health risk assessments [39,40]. Despite these pressures, it wasn’t until the passage of Food Quality Protection Act (FQPA) in 1996 that the EPA was statutorily mandated to consider cumulative risks, albeit from pesticide residues on foods. Still, concerns continued to mount that risk assessment practices were inadequate and failed to address the totality of cumulative health risks associated with real world exposures to a diverse and dynamic combination of both chemical and non-chemical stressors [41].

By 2003, EPA published the Framework for Cumulative Risk Assessment (Framework) on how to plan a cumulative risk assessment, providing a basis for the development of future guidelines promoting consistency in cumulative risk assessment across EPA programs and offices [35]. Although the Framework continued to focus on traditional risk assessment (e.g., summation of individual chemical cancer risks), it emphasized that qualitative analysis of combined effects may be acceptable in cases of limited data or information. In response to the Framework, the National Environmental Justice Advisory Council (NEJAC) and the NRC called on EPA to evaluate combined health risks from multiple environmental stressors, incorporating non-chemical stressors and characteristics of population vulnerability into the agency’s risk assessment process to more accurately capture population risk [5,10]. It was expected that full EPA guidelines on cumulative risk assessment would be published soon after the Framework, ushering in a new era of environmental protection. However, these guidelines have yet to materialize and neither have policies to address cumulative risks and impacts beyond the regulation of certain pesticides and development of clean-up standards for hazardous waste sites [25].

### 1.2. Cumulative Impacts in Maryland

The political landscape in Maryland is complex and varied, and residents’ views towards environmental regulation continue to shift over time. A 2014 study by the Pew Research Center indicated that between 2007 and 2014, residents’ political affiliation remained stable, with more than 50% of adults identifying as Democrats [42]. Over this same time period, Marylanders’ political ideologies gradually shifted towards “liberal,” even as they increasingly favored smaller government with fewer services, and felt that stricter environmental laws and regulations came at a high cost to the economy. In this context, environmental organizations and advocacy groups in Maryland have taken up the challenge of addressing cumulative impacts through policy change [43,44]. In 2009, for instance, community members in Cedar Heights—a predominantly African American town which had been host to multiple gravel and cement transfer stations and asphalt processing facilities—mobilized to oppose an application for a zoning exception brought forth by owners of a concrete batching plant. Although community members were unsuccessful in preventing this zoning decision, they convinced their state legislator to sponsor a bill (SB0706/HB1210) that required any permit applicants proposing to build industrial facilities within Cedar Heights to submit a cumulative impact assessment to the Maryland Department of the Environment (MDE) before a determination on the permit application was reached. [45]. While SB0706 passed Maryland General Assembly Senate floor vote in 2014, the House Environmental Matters Committee failed to vote the companion bill, HB1210, out of committee.

In 2015, proponents brought forward HB0987/SB0693 which narrowed the scope of cumulative impacts assessment to a specific focus on air quality, and required MDE to conduct a cumulative air impact analysis for industries who applied for an air quality permit [46]. The bill also established a public participation process to accompany applications for air quality permits, and required both environmental and health government agencies to work together to study the negative effects of cumulative impacts. Legislators did not pass the bill, and it was subsequently withdrawn by bill sponsors. Undeterred, in 2016, proponents introduced the REDUCE Act which still focused on air quality permitting in “affected communities,” with the language of the bill shifting from cumulative impacts to environmental justice, centering on community engagement in environmental decision making. This bill also did not pass.

Table 1 provides a brief overview of each bill; Supplemental Table S1 offers a more detailed description of each bill.

### 1.3. Social Explanation for Environmental Controversies and Policy Problems

Although cumulative risks and impacts are global phenomena, policy responses to them are localized and shaped by policy-relevant actors’ understanding and portrayal of their importance. Social scientists have explored social explanations for why certain environmental and public health issues have more resonance than others in the policy realm, and have questioned environmental science and public health scholars’ assumptions about the strong influence of objective reality of environmental exposures on public health outcomes. These scholars have argued that environmental health problems do not automatically attract public attention and reach a level of urgency warranting policy change as the result of objective scientific information; rather, it is how such issues are socially constructed which determines whether they resonate with key decision makers [34,47]. That is, whether environmental health issues invoke solutions, including policy change, may have less to do with the health and environmental impacts of substances, products and activities in any objective, materialist sense than with how supporters of the issue are able to construct frames—ways of portraying and constructing a topic—that attract the attention of key decision makers and established institutions that can then maintain these frames [34,47]. Social constructionist analyses therefore contextualize and critically evaluate policymaking as a contested terrain where competing narratives or frames serve difference actors’ interests in light of other evidence [48].

The way an issue is framed has important consequences because it is often a reflection of the underlying assumptions that guide individual and collective interpretation of particular issues [49]. Framing involves the favorable selection of certain aspects and the simultaneous minimization of others in the process of establishing a framework for discussion around the causes and solutions of an issue [34,50,51,52,53]. Additionally, the presence of multiple frames in defining and addressing particular policy problems may lead to a change in the problem itself [54]. For instance, environmental issues may become framed as health issues through the policy process [55]. This is critical in the policy arena because frames can strongly shape how decision makers come to determine facts and how competing frames may augment the intractability of policy-related conflicts [56,57,58,59]. According to Schön and Rein, “intractable policy controversies” are long standing disputes which cannot be settled by references to facts and evidence alone because the inherent framing of such disagreements includes not only the problem, but how it is to be resolved [60].

Scholars have increasingly used frame analysis to examine conflicts over environmental issues, and some have investigated how such controversies specifically mobilize or preclude policy responses [61,62,63,64,65]. For instance, Lewicki and colleagues analyze the Voyageurs National Park Case and the Doan Brook Case to highlight how frame analysis provides a richer understanding of the divisive nature of environmental disputes within public policy [66]. This scholarship illustrates how frames are not only diagnostic (i.e., problem identification and attribution of causality), but also serve a prognostic function by suggesting specific courses of action to bring about solutions [67]. Davis and Lewicki note that there are several core tasks—such as defining issues, formulating action, invoking notion of rights, justifying actions, and mobilizing or precluding collective action—which characterize collective action frames used in environmental and public health conflicts [66]. Policy-relevant actors often use these tasks to develop frames to generate consensus and strategies for action. Disputes in this process can emerge if the definitions of environmental and health conditions or solutions to mitigate their effects are incompatible [68].

We use Maryland’s ongoing legislative process as a case study to examine how stakeholders in intractable environmental health disputes engage in the framing process to bring credibility to their points of view and to discredit opposing perspectives, often overlooking or devaluing information that does not fit within their specific framing of the context. We contribute to the literature by examining how deeply held social values about the association between environmental hazards and public health, functional role of government institutions, and translation of social justice principles into public policy can result in predominant frames related to the lack of data and assessment that have significant effect on the development of policy responses to cumulative impacts.

## 2. Methods

### 2.1. Data Collection

We conducted 35 key informant interviews, long-term participant observation, and content analysis on oral and written testimony presented at state-level legislative committee hearings for the three cumulative impacts bills to gain insight on the perceptions, conceptualizations, and scientific and social framings of cumulative impacts. The University of Maryland’s Institutional Review Board approved all study procedures.

We employed both purposive and snowball sampling to recruit study participants for interviews. We first identified study participants from the legislative record for the three proposed Maryland General Assembly bills, and asked them to participate in in-depth interviews. These participants then referred us to individuals who were knowledgeable about and participated in the development of cumulative risk and environmental justice policies in Maryland. Our aim was to understand the perspectives of policy-relevant actors, rather than to seek a representative sample of the public. Thirty-five individuals representing state legislators, state environment and health agency staff, environmental advocates, business, trade association, and labor union representatives, and community-based advocates participated in interviews lasting 60 to 90 min, from June 2017 to September 2018. Of the 35 participants, 24 provided oral and/or written testimony at one or more of the committee hearings, three represented organizations that provided testimony, and eight otherwise participated in or were knowledgeable of cumulative risk and environmental justice policies at state and federal levels. Table 2 summarizes key characteristics of participants who we interviewed.

Semi-structured interviews were audio recorded and transcribed verbatim. The interview guide was developed from extant literature on cumulative risk and impact, language from the proposed legislation, and our previous research. Interview questions explored participants’ perspectives and experiences of cumulative impacts and risks; barriers and limitations to addressing cumulative impacts and risk; policy environments for environmental health; and relations between various stakeholders working on environmental health issues. Table 3 illustrates sample questions.

All three authors were involved in conducting participant observation and writing field notes after observations throughout the study period. This included visits to key pollution sites included in the three cumulative impacts bills, as well as attending six community meetings related to cumulative impacts.

Further, we transcribed over six hours of video recordings of the bill hearings and photocopies of 465 pages of written testimony from the General Assembly of Maryland’s Department of Legislative Services Library.

### 2.2. Data Analysis

Interviews, along with written and oral testimony transcripts, were compared with the original voice recordings for completeness and accuracy. Field notes were also analyzed. The second and third authors coded four sets of transcripts (i.e., interviews, participant observation field notes, oral testimony, and written testimony) using a thematic approach to data analysis using NVivo 10. We employed a modified grounded theory approach, following the constant comparative method of coding, analysis, and re-coding based on inductive reasoning. Initial broad coding was guided by literature on cumulative risks and impacts and interview guide, with new codes developing from the data [69]. We then used axial coding to make connections between codes, and selective coding to develop major themes [70]. All three authors engaged in routine debriefing sessions to thoroughly discuss and review transcripts, and wrote excerpts and analytical memos to identify emergent themes and differing views to fine-tune analysis that ultimately converged on a shared interpretation. For simplicity in presenting results, we use “participant” to denote both individuals interviewed for our study and individuals who submitted written or oral testimony at bill hearings. For the later, we indicate the bill number. Participants are also identified as “proponents” and “opponents” as indicated in the Maryland General Assembly proceedings; we extrapolate these categories used within the policy process and in participant interviews to designate those who support the three cumulative impacts bills, and those who do not.

## 3. Results

We identified three frames about cumulative impacts as a health issue through which conflicts over policy reforms played out among policy-relevant actors: (a) perceptions of evidence, (b) interpretations of social justice, and (c) expectations of authoritative bodies. These frames emerged from the interview data and were further reflected in participant observation of local sites which were the targets of legislative proposals and community meetings related to cumulative impacts, and content analysis of legislative committee hearings for the three cumulative impacts bills (see Table 4). Below, we illustrate how, under what conditions, and through which processes policy-relevant actors garnered support for their positions, gained acceptance for their problem definition, persuaded other actors to perceive the problem in the same manner, and to dismiss conflicting frames.

### 3.1. The Politics of Evidence: “The Science Isn’t Quite There Yet.”

Policy-relevant actors commonly mobilized various forms of evidence—definitions, health data, and methodological tools—throughout the policy process. They viewed the absence of a shared, distinct understanding of cumulative impacts as a public health issue as a significant barrier to creating public momentum for government action. Participants’ definitions and understandings of cumulative impacts varied widely. Some participants defined cumulative impacts as an environmental hazard, referring specifically to the pollutant load resulting from the combination of emissions from multiple industrial facilities that exist as a result of the state’s permitting process, “My understanding of cumulative impacts is that it’s looking at, for a certain geographic area, not only the impact of a single permitting facility but also all the emitting facilities in a certain geographic area to a community that’s affected” (Participant, Academic Researcher). Others framed cumulative impacts distinctly as a health issue referring to the health impacts resulting from exposure to a combination of physical, chemical, and psychosocial stressors:

*The term cumulative impacts refer to the concept that individuals are exposed to a number of chemical and non-chemical stressors through the course of a day and a lifetime. As you breathe, you may be inhaling large amounts of particulate matter, pesticides, and ozone in addition to the oxygen you need to function. Those are the chemical stressors. You may also be dealing with financial stress, or difficulty with a pre-existing health condition, which also affects your ability to respond to additional stressors you may face. Those are the non-chemical stressors* (Participant, Environmental Nonprofit Representative, oral testimony for SB0693).

Further, during legislative hearings, policy-relevant actors from environmental and health nonprofits and community-based organizations used the terms “health risk,” “at risk,” and “reduce risk” in reference to cumulative impacts as a public health issue. During the interviews, participants were uncertain about the difference between cumulative impacts and risk; yet despite this uncertainty, a majority stated that the notion of risk underscores the probability of something occurring, while impacts indicate something that has already occurred, reflecting broader debates about the associations between environmental pollutant exposures and public health. A participant, a State Government Official, explained, “Impacts are more certain thing so proving with certainty there are actual impacts versus there’s potential risk from the activity.” In some instances, the difference between the terms ‘risks’ and ‘impacts’ indicated geospatial and jurisdictional dimensions, “I think using the term impacts is more localized. It’s a more localized term than risk. If you’re a national entity, it’s a risk because you’re not dealing with the actual impacts. The states and the local jurisdictions are dealing with the impacts” (Participant, Business Representative).

Interview participants noted that regardless of how cumulative impacts were framed--either as an environmental or a public health issue—it was a difficult concept to grasp, making it “hard to mobilize people” to support legislative or policy proposals. A policy maker maintained, “You start talking about cumulative impacts, people’s eyes glaze over. It takes thirty seconds to a min to explain generally what cumulative impacts are it’s not conducive to motivating people to get involved” (Participant, State Legislator). A community advocate agreed, “It’s not easy to develop a process to consider cumulative impacts. But a major barrier is that legislators don’t know how…they don’t understand” (Participant, Community Activist).

Second, policy-relevant actors held divergent perspectives about whether scientific evidence and assessment methodologies to address cumulative impact and risk as a public health challenge had been adequately developed. Bill opponents, in particular, argued that while they deeply understood the urgency of acting on cumulative impacts, a lack of a common approach to address and assess cumulative impacts precluded their buy-in. A participant, a business representative, explained:

*There was some disagreement on the science and methodologies. We didn’t have a consistent agreed approach on how we’re measuring things, and the science behind it, and the real impact the legislation would actually have. If we go and do this, are we going to see the desired impacts that this legislation is claiming that it will help?* (Participant, Business Representative)

Bill opponents framed the absence of a standardized risk assessment approach as a lack of scientific evidence and measurement tools. They argued that insufficient data and analytical frameworks left them without the means to adequately make policy decisions on cumulative impacts, “I think that it’s difficult to write good legislation in the absence of good data and good techniques and methodology” (Participant, State Government Official).

Influential institutional representatives reinforced and maintained this framing, expressing concerns about state agencies’ ability to provide evidence for policymakers to consider:

*There is a very good science on cumulative exposures and the impacts of cumulative exposures. What’s missing is really understanding what the magnitude of that impact...how to measure the impacts, and some of the things that are really complicated are how we can figure out for people who have this lifetime of exposures as well as socioeconomic stress, how to quantify all of that in a way that makes it possible to do what the bill is seeking to do* (Participant, State Government Official, oral testimony for SB0693).

Bill proponents, on the other hand, responded by citing existing federal and state examples to counter opponents’ frames regarding the lack of methodologies for assessing cumulative risks or impacts. During the SB0693 hearings, an academic expert argued, “There’s a lot of screening approaches that are being used to look at exposures, look at burden, look at environmental effects, look at public health effects, and look at both vulnerability and susceptibility. The screening tool is being used right now in California with their cumulative impacts methodology. It’s being used in Minneapolis, Saint Paul with their cumulative impacts approach as well” (Participant, Academic Researcher, oral testimony for SB0693).

Community stakeholders also argued that existing assessment strategies were sufficient for enacting policy measures. One participant who was a member of the Maryland Commission on Environmental Justice and Sustainable Communities, during hearings on HB1210, maintained: “The U.S. Environmental Protection Agency has spent the last ten years working on developing these tools. They right now have seventeen different screening mechanisms, C-FERST, EJSEAT, and a whole pantheon of different tools that allow facilities, that allow states and regulators to measure and look at what the differential exposures are to communities” (Participant, Community Advocate, oral testimony for HB1210). Overall, participants’ views about the construction of cumulative impacts as a plausible public health issue varied, with bill proponents and opponents holding conflicting perspectives about the existence of credible evidence to advance policy on such matters.

### 3.2. The Promise and Peril of Environmental Justice: “I Understand Clean Air, Clean Water, But We’ve Gotta Have Jobs”

From the outset, bill proponents framed cumulative impacts as resulting from environmental and health injustices, demanding that policy makers and government agencies take responsibility for remediating such inequities among low-income and racial and ethnic minority populations. In the first bill, SB0706/HB1210, cumulative impacts was framed as an environmental health problem that, “prevails in communities that are primarily minorities and poor people” (Participant, State Legislator, oral testimony for SB 0706), and focused on getting the Maryland Department of the Environment (MDE) to conduct a cumulative impact assessment and make it available to the county planning and zoning authority and to the public before preparing determinations on permit applications.

Environmental and health justice frameworks were consistently used in each of the cumulative impacts bills but became more explicit in the second and third bills introduced in 2015 and 2016, “We brought those indicators [from the Maryland Health Improvement Health Disparities Reduction Act] into a revised version of that bill. The whole idea was we wanted to have more focus on environmental justice, and how environmental justice impacts health” (Participant, Academic Researcher). Bill proponents, in many ways, conceived of cumulative impacts as means to advance policy addressing environmental and health injustice, “What was clear to me over these last few years is that the term environmental justice needs to be put into legislation. I think cumulative impact is terribly important to understand because it gives us the basis to fight” (Participant, Community Activist).

After the first two bills failed, the third cumulative impacts bill, framed entirely through an environmental justice framework, underscored that an increased role for local, community voices, combined with an environmentalism more responsive to economic injustices, would tend to be protective of public health among affected communities. When introducing the REDUCE Act, a bill sponsor acknowledged that proponents, “decided to take a step back from last year and look a little bit more closely at what we can do to help improve notification and awareness of local communities” and that he would not consider the REDUCE Act to be a cumulative impacts bill, but rather “better information disclosure for communities to make them aware of what’s being built nearby” (Participant State Legislator, oral testimony for HB0820). A community advocate asserted:

*I’m here because I see in my own community and the communities surrounding it a lack of environmental justice. I see new industry piled upon existing industry. I see new pollution in communities that are already overburdened. Tractor-trailers are constantly present on residential streets of our neighborhoods. They wake us in the morning, and they interrupt our sleep at night. They rumble past our houses, community center, and our new high school. They ruin our roads, and they spew pollution into our air. But this is not about the technical issues of cumulative impacts. It’s not even about diesel truck traffic per se. What it is about is public health and fairness. It’s about giving underserved communities a voice* (Participant, Community Activist and member of the MDE Cumulative Impacts Workgroup, oral testimony for SB0398).

The focus for bill proponents was on elevating distinctive community voices whenever possible in the policy making process. A community activist stated at the first hearing for the REDUCE Act, “Environmental justice calls for fair treatment, having a seat in the room within the decision making process” (Participant, Community-Based Organization Representative, oral testimony for HB0820). Several participants noted that communities often became aware of permit decisions only after they occurred:

*That was one of the things we tried to do, and that bill figured out a trigger much earlier in the process for community outreach and engagement so communities can come to the table* (Participant, Environmental Nonprofit Regional Director).

Community engagement requirements in the REDUCE Act were seen as bolstering affected communities’ capacities to “learn more about those important connections between environmental and health and ensure that those who are affected are involved in the decision-making process” (Participant, Environmental Nonprofit Director for community outreach, oral testimony for SB0693). Bill proponents argued that impacted citizens, when armed with information about cumulative impacts as a public health issue, could become advocates and ultimately improve the [permit] decision-making process overall, “At the very minimum, you want to make sure that people have a level playing field with respect to information and data. That’s one thing you can do, even if you can’t give them necessarily equal power on the basis of money or other factors” (Participant, State Government).

However, as a shift in framing occurred over time to include “protected communities” and “affected communities” explicitly defined by socioeconomic, demographic and health status indicators (e.g., Medicaid enrollment, low birth weight rates, poverty rate, percent minority), bill opponents increasingly mobilized frames that reinforced the lack of causal associations between multiple environmental pollutant and social stressor exposure and health outcomes. For instance, they argued that cumulative impacts could be determined by multiple issues, especially individual behaviors and lifestyle factors. A government representative supported and maintained this framing:

*There’s poverty levels, and that’s where I think with cumulative impacts we struggle because there’s so many factors that contribute to people’s health. It’s whether you exercise, it’s what your diet is, it’s what your levels of stress are, it’s whether you smoke, your behavioral factors and things like that. And then on top of it you add some sort of environmental pollution…environmental pollution just icing on the cake to all these other things* (Participant, State Government Official).

They also argued that the health of affected communities was significantly impacted by economic challenges, “When I see somebody that’s making $25,000 or less, and they have a family of four, what are they going to do when they go grocery shopping? They’re going to look for shortcuts. They probably don’t have the best medical plans” (Participant Labor Union Representative, oral testimony for SB0693). They framed the bills as measures that could potentially further harm impacted communities in hindering much needed economic development, “Obviously our interest is we don’t want our workers to be harmed, and we certainly appreciate environmental justice, but we also think that economic justice should be of value as well” (Participant Labor Union Representative, oral testimony for HB0987). Similarly, another participant used economic justice to frame solutions, “You’ve got to have jobs. You can’t blame all your health issues on a smokestack. If you don’t have economic justice, people’s health is going to be impacted” (Participant, Labor Union).

As the bill language and legislative session discourses shifted more specifically towards environmental justice, opponents framed the proposals as “vague,” “too broad,” and “unclear.” For instance, a participant argued:

*The lack of standards, if you look at the legislation, there’s no standards or criteria that [unclear] what this impact analysis would include. We have a lot of concerns about how long that type of analysis might take, what types of twists and turns it would take, the way it would undermine the predictability of going through the permit process which is very specific in nature, has very specific criteria directed at protecting the resource that’s being impacted or that is a part of the development process* (Participant, Trade Association Representative, oral testimony for HB0820).

Calls for increased community participation in the decision-making process were portrayed as redundant and burdensome on existing regulatory procedures:

*There needs to be an opportunity for community input, that’s a legitimate issue, you’re going to build a brick factory in my backyard I ought to have an opportunity to at least know something about that, and voice my opposition if you’re going to discharge your affluent into my backyard. Okay, I understand that, but how many bites of the apple should those groups have an opportunity to take? Our point of view is too many opportunities, because every time you inject a lawsuit, that permitting process just stretches out and stretches out. That is a tactic of the environmental groups, and I’m not saying they’re bad human beings, but that’s a tactic of the environmental groups. I don’t think...we don’t think it serves the public as well as a more efficient process could* (Participant, Trade Association Representative).

Bill opponents also depicted the shift towards promoting community input and involvement as moving focus away from more “scientific” and “evidence-based” factors related to cumulative impacts such as environmental and health risks. A participant noted, “*I think it’s an environmental justice bill, making communities that are nearby to these facilities much more aware of what’s coming in, but it doesn’t allow MDE to prevent these permits from being issued. It avoids those complex modeling and evidence and data that you had cited for cumulative impacts, so I don’t think this is a cumulative impacts bill*” (Participant, State Legislator, oral testimony for HB0820). Throughout the policy process, bill proponents and opponents’ frames remained incompatible in the translation of social justice principles into public policy.

### 3.3. The Dilemma of Authority: “Unfortunately Our State Agencies Are Not Doing Enough to Work Together”

Each of the proposed bills outlined requirements and responsibilities for state-level environment and health agencies. Despite acknowledging that cumulative impacts disproportionately affected the health of low income and minority communities, government agencies testified in opposition to the bills, sustaining the frames developed by bill opponents. State agency representatives, along with industry advocates, framed their position as stemming from lack of expertise, capacity, and authority. Policy-relevant actors who testified in opposition, for instance, noted that the proposed bills would change current permit approval practices in such a way that it would demand different analytical skills and may conflict with or be different from existing federal procedures, “This [bill] is a major change in the standard of review, requires the Department to do things that are beyond its technical capabilities” (Participant, Trade Association Representative, oral testimony for HB1210). Others cited constraints of existing regulatory infrastructure for permitting or pointing to land use and zoning agencies as the real causes of cumulative impacts and thus should be the targets of any new policy instead:

*The [health] department is in opposition to the bill. The primary concern here is that this is a very complicated and new undertaking for the department. It’s a process that might involve some 30–50, we’re estimating, permit reviews a year. Part of the challenge is we [health department] don’t live in the air permitting business, we live in the health business. It’s not something the department has previously done and it would require a fair amount of work* (Participant, Stage Government Official, oral testimony for HB0987).

State environmental agency staff reinforced this framing by stating that they only have the capacity and authority to follow current federal EPA guidelines:

*We don’t make that public health decision directly, but the feds have already done that. In none of our air permitting requirements do we look at direct impacts to public health...we review the concentration of those emissions. It’s not cumulative, just that particular one source, and gauge it against set criteria that federal toxicologists have developed for worker safety levels. We divide by 100 to make sure that there’s a threshold, a safety factor for kids and for sensitive populations. If with that math you’re below that federal level, then you pass. That’s about the only public health link we have to what we do* (Participant, State Government Official).

Bill proponents, meanwhile, attributed the problem of cumulative impacts to a lack of accountability. Community stakeholders and legislators who were in support of the bills framed industry and government agencies as those who did not want to act on cumulative impacts because they failed to take responsibility for ensuring the environmental safety and well-being of impacted communities. A local legislator testified, “We’ve attempted to have some conversation with the owners of the facilities to try to resolve some of these issues, but we feel it is pertinent for the state to step in and to assist in addressing this issue that we feel is of grave concern to the community” (Participant, State Legislator, oral testimony for HB1210).

Bill proponents also expressed frustration with what they perceived to be the inherent fragmentation of governing bodies. Participants explained that state agencies lacked the ability to work together to protect communities. An academic researcher stated, “Unfortunately, our state agencies are not doing enough to work together, like MDH and MDE” (Participant, Academic Researcher). A community advocate supported this sentiment, “Go to MDE and they say ‘Well, we’re following our procedures, and our procedures say we have to approve this.’ Go to the county council and they say ‘Well, it’s zoned already,” and even if it’s not zoned they’ll give a pass to it, because they have an exception rule” (Participant, Community Activist). In the context of the three Maryland cumulative impacts bills, participants’ framing of who is accountable and has authority over regulating cumulative impacts as a public health issue diverged widely.

## 4. Discussion

The current study examined policy-relevant actors’ perspectives on the challenges related to advancing policies to reduce cumulative environmental human health impacts in Maryland using ethnographic research methods and frame analysis grounded in a social constructivist framework. To our knowledge, this is the first study to draw on constructivist and interpretive approaches to better understand the implicit role that values, beliefs, and worldviews have on the cumulative impacts policy process. Framing—the dynamic process whereby individuals make sense of ideas by interpreting them through available social concepts and principles—mobilizes support for certain perspectives over others and can drive policy in a particular direction [59]. If we consider how framing affects each stage of the policymaking, we can better engage in understanding the influence of ideas and motivations in the policy process.

Our findings illustrate that policy-relevant actors utilized multiple, conflicting frames to define problems, determine causes, make moral judgments, and propose mitigation measures related to cumulative impacts. These frames determined what policy-relevant actors considered to be legitimate “facts” about the associations between multiple environmental pollutant and social stressor exposures and human health effects and how competing perceptions of the problem of cumulative impacts influenced normative measures for action. Social justice principles--focused on the value of community participation and knowledge about the inherent linkages between cumulative impacts and population vulnerability—were framed as less credible than more “scientific” forms of evidence—data, metrics, assessment tools. Even when bill proponents attempted to strategically reframe cumulative impacts bills to incorporate a broader array of interests around environmental justice and potentially overcome the stalemate in the decision-making process, conflicting frames around problem definition and solutions thwarted policy action.

Specifically, participants cited a lack of a common definition and evidence, including data, methods, tools, and frameworks as key challenges in advancing policy on cumulative impacts in Maryland. Policy-relevant actors, both bill proponents and opponents, understood cumulative impacts and risk to mean a combination of pollutant emissions and social stressors that have the potential to or does impact human health. Policy-relevant actors agreed on the material reality of cumulative impacts and risks, yet also conceded that the lack of a common definition as well as its conceptual complexity further complicated how it became understood and interpreted as a health issue in the policy arena. Even when bill proponents shifted the frame more concretely toward environmental justice in the last two bills, they had little success in garnering support from key decision makers. Bill proponents’ claims that cumulative impacts bear a disproportionate burden on low-income and racial minority communities, and that health effects associated with environmental pollutant exposures have also been greatest among these communities had little resonance with bill opponents.

In response, bill opponents, both policy-relevant actors and regulatory institutions, framed cumulative impacts as an inherently complex issue, arguing that the compound nature of cumulative impacts made it difficult to tease apart its disproportionate health impacts on vulnerable communities. For instance, they attributed health disparities as stemming from structural challenges related to economic deprivation, arguing that stricter environmental regulation would deter job creation and essential economic development in communities with the greatest need. Further, opponents viewed the shift in the bill language, from cumulative impacts to environmental justice, as an indication of proponents’ inability to clearly define cumulative impacts as a public health issue and pursue a coherent set of environmental health policy priorities. They portrayed environmental justice frames as “tactics,” solely focused on elevating community voices rather than those which sought to redress environmental health risks through evidence-based practices.

In addition, bill opponents framed the lack of evidence and clear legal authority to assess and address cumulative impacts as rationale for not taking policy action. Influential institutions, including state regulatory agencies, maintained these frames, noting that if more robust data and scientific tools are developed, policy action should follow. Bill proponents countered by referencing other states that have addressed cumulative impacts in permitting through legislation (e.g., MN and CA) as well as the panoply of assessment tools available through EPA. These examples had little resonance with bill opponents and policy makers.

While our study focused specifically on cumulative impacts legislation in Maryland and local experiences of policymaking, contested meanings over knowledge and evidence have been central issues documented by the scholarship on intractable environmental health policy controversies in other settings. This suggests a broader significance of our findings. For example, bill opponents’ framings of a lack of evidence, analytical tools, resources, and clarity in legal authority reflect what some scholars have called a “standard narrative” in previous research analyzing the slow progress of policy implementation to address environmental justice, cumulative environmental health risks and impacts, and health inequities [12,71,72,73]. Rooted in this standard narrative, the vast majority of environmental health sciences research on cumulative risks and impacts calls for the development of more fined-tuned assessment methods and data for use in the regulatory context, assuming that these efforts will lead to better policy decision-making [11,12,15,17]. A number of screening or surveillance tools to identify “overburdened” communities exist, and such mapping may help guide prioritization of local level environmental health interventions and investments to protect disproportionately exposed or vulnerable populations [26,27,74,75,76]. Yet, the almost exclusive institutional focus on developing mapping algorithms and other risk assessment methods as solutions to “cumulative” contributes to disagreements over how to define “overburdened communities,” what forms of evidence and expertise are considered valuable, trustworthy and credible in policy deliberations that attempt to frame environmental issues as public health challenge, inducing a type of “paralysis by analysis,” where the process of attempting to assess risk significantly slows down or even prevents government interventions [10,16,77,78].

While we don’t deny that analytical advancements and clarity in legal authority may boost the capabilities of government agencies to act on cumulative risk and impacts, the near exclusive investments by environmental health experts and governmental research institutions into tools, data, and methods on chemical mixtures and cumulative risk [11,12] have not led to widespread mobilization or action in the policy arena. Even in instances where policy advances have been made on cumulative impacts such as California, New Jersey, and Minneapolis, regulation and implementation has proved to be difficult and complicated, and where debates over evidence, social justice, and authority continue to intensify [26,29,77,79,80,81,82,83,84,85,86,87]. Our findings indicate that this focus on particular forms of evidence highlights overtly and implicitly certain aspects of the problem, while obscuring other impediments to policy actions to mitigate cumulative environmental health risks and impacts.

For instance, we found that policy-relevant actors’ framings of “affected” communities—largely low-income and racial minority populations—and racial disparities in health also hindered policy progress on cumulative risks. Bill opponents contested bill proponents’ claims of disproportionate pollution impacts and discriminatory regulatory enforcement by framing cumulative impacts as also stemming from inherent individual behaviors, lifestyle factors, and economic inequalities of communities themselves. Scholars studying conflicts over regulating toxic chemicals, use of risk assessment in policymaking, and the incorporation of environmental justice into regulatory activities at federal and state agencies have also documented similar frames which focus on individual responsibility alone. Such frames shift attention away from policies and institutional factors that systematically create and maintain racial and socioeconomic disparities in health, and have the potential blame individuals and communities for poor health outcomes [71,72,88].

Although environmental decisions by industry and government are widely recognized as having potentially serious equity implications, especially for low-income and minority communities, empirical support for environmental health disparities is commonly understood as inadequate or imprecise by those in the policy arena, for whom disproportionate pollution impacts on health outcomes are framed mainly as a result of behavioral and cultural factors, and to a lesser degree structural factors such as economic inequalities and access to health care [71,89]. As Jill Harrison notes in her study of federal environmental justice policy implementation, such framings of disparate health outcomes among low-income and minority populations are reinforced by social norms within regulatory agencies to “maintain a level playing field for industry” [71]. Any regulatory reforms that would result in imposing different or more stringent standards to some industrial operations “just because of socioeconomic problems in some areas but not in other areas” violates agency’s “bureaucratic neutrality” towards the regulated community [71].

Further, our findings highlight that the ideology of environmental justice as a policy initiative may limit legislative measures on cumulative impacts. Proponents increasingly came to view cumulative impacts bills as opportunities to legislate on environmental justice issues. Such shifts in bill proponents’ frames underscored direct citizen action and influence as legitimating forces in policymaking, rather than scientific expertise and institutional interests [90]. The focus of the REDUCE Act, the final cumulative impacts bill, centered more on advancing community input and enabling citizens to take action to protect their own health than on curbing environmental hazards and health risks. Such framing deviates from longstanding priorities of environmental justice and aligns with bill opponents’ neoliberal and individualism frameworks (e.g., if citizens have good jobs with good money, they can choose to live elsewhere and thus could limit their exposures).

Although the environmental justice movement has historically focused on pursuing stronger regulatory restrictions on environmental hazards, scholars have noted shifts in both environmental justice programming within governmental agencies and among environmental justice advocates towards more individualistic actions as mechanisms for change such as educating residents about healthy lifestyles, building green spaces and other environmental amenities, and promoting better communications between residents and polluting facilities in their communities [72,91,92]. The shift in bill proponents’ frame also parallels a neoliberal shift within the broader environmental policy and regulatory system evidenced by deregulation, the embracing of market-based and voluntary solutions, and shifting regulatory responsibility to local governments and even to individuals (e.g., by becoming informed consumers we can protect ourselves by avoiding chemical exposures [24,71,93,94]. Interventions to address cumulative impacts, when framed through an environmental justice framework that has shifted toward neoliberal concepts of individual responsibility (e.g., emphasizing individual choice without commitment to remove or lessen environmental hazards in the community), has the potential to direct community and institutional attention away from issues presenting the greatest risk to community health and further exacerbate the continued impasse in environmental policymaking.

Finally, our findings highlight how the current stalemate over cumulative impacts legislation reflects broader debates over governance and authority in the policy realm, and the increasing decentralization of the policy process. At both the federal and local levels there are multiple governing agencies, including federal agencies that operate through national, regional, and state-level offices, each charged with substantial discretion to oversee some aspect of environmental health protection, but they rarely work together [95,96]. Such institutional arrangements derive from and continue to coalesce with industry interests and activities. Both the Maryland state health and environmental agencies upheld bill opponents’ frames despite acknowledging that cumulative health effects of multiple environmental stressors were likely occurring, particularly among minority and economically disadvantaged communities. They framed their opposition around their authoritative function and statutory and regulatory constraints vis à vis the federal government, emphasizing the lack of federal direction on addressing cumulative impacts and their desire to stay within their agency mandates to address either environmental or health issues, rather than both. Some scholars have used the concept of “boundary work” to underscore this phenomena where governmental agencies defend the status quo against calls for change [71,97]. State agencies resisted the reforms proposed by the cumulative impacts bills by asserting conflict with the identity of the organization (e.g., “we don’t live in the air permitting business”) and drawing disciplinary boundaries between public health, environment, and land use planning.

Additionally, a review of state environmental health infrastructure underscores that the mandates of federal environmental laws has forced a narrow focus of state agencies towards the technical aspects of environmental protection and away from broader public health aspects, while budgets and staffing levels have been greatly reduced [95,98]. This has implications for state capacity to take on complex dynamic environmental health issues that require cooperation between many agencies and innovation. We argue that this portrayal of the limited role of governmental agencies has implications for other proposed state-wide and national-level initiatives that require cross-sectoral collaboration such as “health in all policies” and those which address climate change. Understanding these frames are areas for future research.

## 5. Conclusions

The combined effects of multiple environmental toxicant and social stressor exposures are widely recognized as important public health problems, likely contributing to health inequities [3,6,7]. Since the 1980s, environmental justice advocates, stakeholders, and independent advisory bodies have called for federal and state environmental agencies to use cumulative assessments in regulatory and policy decisions [5,10,38,41,99,100]. However, there has been very little progress by state and federal agencies on developing comprehensive strategies to mitigate and prevent cumulative environmental health risks and impacts. Previous analyses on the challenges to regulating cumulative risk have mainly focused on a lack of data, analytical tools, and methodologies [12,15,17]. While these material factors are important inputs, it is not the whole story.

Our findings demonstrate that by framing ideas about cumulative impacts and risks in certain ways, policy-relevant actors draw on deeply held social values about evidence, social justice, and authority that transform environmental hazards into contested health policy arenas. Divergent ideas regarding the credibility of particular forms of knowledge and evidence about cumulative impacts of exposures to multiple environmental pollutants and social stressors arose during the policy process, and bill opponents’ framing of a “standard narrative” held more traction with policy makers. Even when proponents reframed the cumulative impacts bills to incorporate a broad array of environmental justice considerations to gain political support, these frames proved incompatible to policy progress. Environmental justice frameworks focused on community knowledge of disproportionate burden of environmental hazards and health risks and participatory governance were perceived as less credible than more objective, scientific forms of evidence. These issues underscore long standing conflicts over governance, authority, and related constraints of decentralization in addressing environmental health challenges.

By examining how policy-relevant actors framed the relevance of cumulative impacts and risks, we illustrate how individual perspectives and institutional norms about evidence, authority, and environmental justice function to stall progress to legislate on cumulative risk and impacts. We argue that the discursive techniques used by policy-relevant actors and regulatory bodies to frame cumulative impacts in terms of impacted communities, health disparities, and social justice significantly shape legal and regulatory outcomes in policy realms.

Our study adds to the body of literature on cumulative risks and impacts by using interpretive and ethnographic approaches to analyze environmental policymaking as a contested meaning-making process. It also extends framing theory in policy studies by providing insight into how and why proposed environmental health policies are supported or dismissed.

Our work has several implications for future planning and research agendas related to cumulative risk and impacts policymaking. Studies that focus on how ideas and policies around cumulative risk and impacts become framed are essential for understanding longstanding policy impasses at the federal and state levels. Analysis of social forces which shape policy, and in particular, the role of ideas, motivations, and behaviors of policy-relevant actors should also be prioritized, especially those that identify and examine frames, framing processes, and frame conflict within intractable environmental health policy controversies. Social scientists, including ethnographers, have been well-recognized as critical public health researchers because of their ability to assess social, economic and political factors in local and global contexts. Interdisciplinary work on cumulative impacts, involving social scientists, environmental health scientists, activists, and policymakers, has the potential to generate considerable insight into the nature of policy debates in environmental health, including drawing from successful policy processes and social movements. Specifically, anthropological and ethnographic findings can illustrate why policy proposals succeed or fail: the divergence between what is proposed and what is attained on the ground and the unplanned outcomes that may result. These research collaborations can also aid proponents of cumulative impacts policy reforms in bringing greater focus to complex, underlying debates related to the relations between environmental hazards and their public health impacts, the functional role of governing bodies, and translation of social justice principles into public policy that will need to be addressed if they hope to be successful. Finally, inclusive partnerships between environmental health scientists and community partners should be intensely considered, as they have the potential to build alliances to challenge institutional and policymaking norms that keep intact frames that prevent policy progress.

## Figures and Tables

**Table 1:** Evolution of proposed cumulative impacts legislation in Maryland 2014–2016.

HB1210/SB706 Environment-Permit Determinations-Cumulative Impact Assessments 2014	HB0987/SB0693 Cumulative Air Impact Analysis 2015	HB0820/SB0398 The REDUCE Act-Reducing Environmental Degradation for the Underserved through Community Engagement 2016
This bill requires permit applicants to submit to Maryland Department of the Environment (MDE) a cumulative impact assessment before preparing a tentative determination on an application for permits for operations in unincorporated communities in Prince George’s County. The assessment must address the likely impact on the environment and on human populations that will result from the incremental impact of the activity or proposed facility authorized under the permit when added to the impact of other past and present sources of pollution. MDE must provide a summary of the results of the assessment to the Prince George’s County planning and zoning authority, and, for a specified air quality permit, must post the results on its website. MDE may adopt regulations to implement the bill.	This bill requires MDE to conduct a Cumulative Air Impact Analysis (CAIA) upon receipt of an application for an air quality permit to construct in a “protected community” in the state. If MDE concludes, following a CAIA, that the proposed activity will have an impact, MDE is required to take specified actions on the permit, potentially including denial of the permit. The bill establishes a public participation process to accompany applications for air quality permits, and requires MDE and the Maryland Department of Health (MDH) to study the negative effects of cumulative impacts of pollution and other topics. MDE may adopt regulations to implement the bill.	This bill requires applicant for an air quality permit to (1) estimate and report specified information related to diesel vehicle trips and emissions to MDE and (2) solicit specified information from an “affected community” located around a source or proposed source. “Affected community” means a U.S. Census tract in which the source or proposed source is located that meets specified income and race criteria. Before issuing such a permit, MDE must (1) solicit specified information from the appropriate county or local health department related to incidences of specified health ailments within the affected community and (2) coordinate with the permit applicant to disseminate the information to interested parties.

Note: See Table S1 for a more detailed comparison of the cumulative impacts bills’ language.

**Table 2:** Interviewed participant characteristics.

Stakeholder Group	Number Interviewed	Context
Maryland General Assembly	2	Members of the Maryland General Assembly’s Environment and Transportation Committee.
Environmental and Health Agencies	8	Members of the EPA, MDE, MDH, and local government agencies.
Businesses, Trade Associations, Labor Organizations	6	Members of Maryland-based businesses and chapters of trade associations and labor organizations.
Environmental and Health Nonprofits	9	Members of national, regional, and state environmental and health nonprofits.
Community-based Organizations and Community Leaders	7	Members of communities and community-based organizations in Baltimore, Baltimore County, and Prince George’s County.
Academic and Research Experts	3	Members of academic and research communities.

**Table 3:** Sample interview questions.

Describe your perspective on cumulative risk. Probe: How have you come to understand cumulative risk? Describe the sources of information that inform your perspective. Describe your understanding of cumulative environmental health impacts? Probe: How do you see relationship between cumulative risk and cumulative impacts? Can you describe the kinds of barriers and limitations to addressing the issue of cumulative risk in Maryland? Describe whether cumulative risk is an environmental health priority for the Maryland State government to address? Why? Who are the main stakeholders involved in environmental health legislation and policy in Maryland? Probe: What are the relations between these different stakeholders?

**Table 4:** Overview of the identified frames and subthemes.

Frames	Subthemes
Evidence and knowledge	Cumulative Impacts
	Cumulative Risk
	Data and Data Collection
	Knowledge Production, Ownership, and Transfer
Social Justice	Economic Justice
	Environmental Justice
	Balancing Economic and Environmental Justice
Authority and accountability	Association Between Industrial Pollution and Health Outcomes
	Mitigation
	Policy Environment
	Experiences with Maryland Cumulative Impact Policy

## Supplementary Materials

The following are available online at https://www.mdpi.com/article/10.3390/ijerph18083947/s1, Table S1: Detailed Comparison of Language and Requirements from Three Cumulative Impacts Bills Considered by Maryland State Legislators.
