# Supplementary material for: Framing Environmental Health Decision-Making: The Struggle over Cumulative Impacts Policy
Source: Int J Environ Res Public Health. 2021 Apr 9;18(8):3947. doi: 10.3390/ijerph18083947 (PMC8070174; doi:10.3390/ijerph18083947)
Supplement: Supplementary file 1 [file ijerph-18-03947-s001.pdf]

Table S1. Detailed Comparison of Language and Requirements from Three Cumulative Impacts Bills Considered by Maryland State Legislators.

	HB1210/ SB706 (2014) **Environment - Permit Determinations - Cumulative Impact Assessments**	HB 0987/SB 0693 (2015) **Cumulative Air Impact Analysis**	HB 0820/SB0398 (2016) **The REDUCE Act -Reducing Environmental Degradation for the Underserved Through Community Engagement**
Method of Assessment	Cumulative Impact Assessment	Cumulative Air Impact Analysis	Estimation of the total number of number of diesel trucks and emissions associated with their operations that will service a source or proposed source during construction and operation
Assessment Details	The Cumulative Impact Assessment is required to be conducted and submitted by the applicant to the Department of the Environment before the Department prepares a tentative determination on a certain permit application. The applicant shall submit the assessment to the Department within a certain timeframe determined by the Department, and the Department shall review the assessment.<br><br>The assessment shall address the likely impact on the environment and on human populations that will result from the incremental impact of the proposed facility or activity authorized under the permit when added to the impact of other past and present sources of pollution.	A Cumulative Air Impact Analysis is required to be conducted by the Department of the Environment and reviewed by the Department of Health Mental Hygiene before the Department of the Environment issues a permit.<br><br>If the permit involves PM, NOx or VOC air pollutants, the Department of the Environment shall require the major source permit applicant to conduct an air sampling plan for at least 12 months before the issuance of a tentative determination on the permit or 6 months for a nonmajor source<br><br>The assessment will determine the air quality impacts of the expected emissions from the proposed activity (permitee), including any mobile source air emissions resulting from the proposed activity and the current air pollutant load for similar pollutants in the immediate area and determine the potential health effects of any air quality impacts.	The permit applicant is required to estimate and reporting the number of diesel vehicle trips per day needed to service the source or proposed source during construction and operation and the associated emissions from the diesel vehicle trips.

		Upon completion of the Cumulative Air Impacts Analysis, the Department of the Environment shall issue a report containing all data relevant to the analysis and all the Department's relevant conclusions.<br><br>The Department of the Environment and the Department of Health and Mental Hygiene will conduct research to identify factors that contribute to the negative effects of cumulative impacts of air pollution and other stressors on a community; review state of the science for environmental justice screening tools; review statewide health and demographic data and update all of these reviews every 5 years.	
Applicable Permits/sources	• Air quality control permits to construct subject to § 2-404 of this article for a new course of a major modification to an existing sources that is subject to 40 CFR§52.21 of COMAR 26.11.17<br>• Air quality permits to construct subject to minor new source review<br>• Permits to install, materially alter, or materially extend landfill systems; incinerators for public use, rubble landfills, solid waste transfer stations or solid waste processing facilities subject to §9-204<br>• Permits to discharge pollutant to waters of the state issues for new industrial facilities in accordance with §9-323(A)	• Air quality control permits to construct subject to § 2-404 of this article<br>• Any general permit issued under to § 2-404 of this article<br>• Any certificate of public convenience and necessity subject to § 2-405 of this article	Applies to the construction of a new source, or the modification of an existing permitted [air] source that makes a physical or operational change to the existing source that will result in a significant net increase in emissions of any pollutant from the source if the source is location in an *affected community*;<br>AND<br>Engages in the following activities:<br><br>• Permits to operate the use of crushers, hammer mills shredders, grinders or classifying screens of 5 tons (4,540 kg) or more per hour throughput

	• Permits to install, materially alter, or materially extend a structure used for storage or distribution of any type of sewage sludge issued in accordance with §9-231<br>• Permits to own, establish, operate, or maintain a new controlled hazardous substance facility issues in accordance with §7-232<br>• Permits to own, establish, operate, or maintain an new hazardous material facility issues in accordance with §7-103 of this article<br>• Permits to own, establish, or maintain a new low-level nuclear waste facility §7-233		• Coal, or ore export loading or unloading installations<br>• Asphalt blowing or asphalt building products, saturation or roll coating installations<br>• Hot-mix asphalt concrete production installations
Determinations	Based on the cumulative impact assessment, the Department can decide to:<br>• Require additional assessment as considered necessary<br>• Issue or not issue a permit<br>• Propose permit limitations or conditions deemed necessary to lessen negative impacts on the environment and on the human populations<br>The Department will provide a summary of the assessment results:<br>• When issuing a tentative determination<br>• For a permit issued under subsection (A)(1)(II), by posting the results of the assessment on the department website for at least	Based on the Cumulative Air Impacts Analysis, the Department of the Environment shall make a tentative determination as to whether the proposed activity will impact the immediate area in one of three ways:<br>• No impact<br>• Some impact<br>• Significant impact<br><br>The Department of Health and Mental Hygiene shall review the Cumulative Air Impacts Analysis report and tentative determination and provide comments and recommendations which must be addressed by the Department of the Environment's final permit determination.<br><br>If the Department of the Environment concludes some	Before issuing an air quality permit to construct for any of the affected activities, MDE must<br><br>• Require the applicant to estimate and report the number of diesel vehicle trips per day needed to service the source or proposed source during construction and operation and the associated emissions from the diesel vehicle trips; and<br>• Solicit input from the affected community and community advisory board or similar organization that is located within a one-mile radius of the source or proposed source on diesel vehicle trip routes and times, impacts on road infrastructure and traffic safety, and idling policies.

	two weeks before issuing the permit<br>The Department will provide a summary of the assessment results to the local government planning and zoning authority in the jurisdiction where the propose activity or facility authorized under the permit will be located, for review and consideration in any future land use decisions	impact for major or nonmajor source or major or nonmajor modifications permit applicants, the Department of the Environment may issue the permit under certain conditions.<br><br>If the Department of the Environment concludes significant impact the Department of the Environment shall:<br>• Deny the permit OR<br>• Issue the permit with terms that prohibit all expected air pollution from the proposed activity and mitigate expected air pollution from mobile sources serving the source.	• Publish that information on its website before issuing a permit.
Public Interactions	The public process laid out by this bill follows current law:<br>• Draft permits will be available to the public<br>• A notice of the tentative determination will be published by the Department and will be open for public comment for 30 days before a final determination is issued<br>• The hearing can be canceled it the persons who submitted written requests withdraw them prior to the meeting<br>• Notice of a final determination will be published if the Department is required to prepare one	The public process will follow what is already in current law and the following:<br>• The Department of Environment will notify all nearby residents, property owners, schools, churches, elected officials by mail; and post notices at proposed source location and public facilities in the area that a cumulative air impact analysis is being conducted and solicit public comment<br>• The Department of Environment will hold public meetings; allow for submission of written comment and will provide written response to comments<br>• The Department of Environment will hold public meetings	Before issuance of the permit for the activities listed, the Department of the Environment will:<br><br>• Upon request by an individual residing within an "affected community" solicit from the appropriate county/local health department information on respiratory illness or mortality; cardiovascular disease; or other health disparities of concern within the affected community from county and local health departments;<br><br>• Coordinate with the permit applicant to disseminate the health information and the pollutant data to representatives of the affected community; local

		• A notice of the tentative determination will be published by the Department and will extend public comment to 90 days and hold public hearing before a final determination is issued	businesses, advocacy organizations known to have worked with the affected community and elected officials of the affected community before issuance of permit
Identifying Environmentally Stressed Communities	The bill only applies when the proposed facility or activity authorized under the permit would be located in an unincorporated community in Prince George's County that:<br>• Is bordered to the north by a US Highway and to the couth by a state highway<br>• Is within 2 miles of a parkway maintained by the National Park Service<br>• Is within 1 mile of a Metro station<br>• Is within 1.5 miles of the District of Columbia<br>• Has experienced air quality alert days of dangerous air quality for sensitive populations<br>• Is located near several heavily trafficked state and county roads that carry both truck and automobile traffic	This bill only applies to "protected communities" defined as zip codes that:<br>• have economic disadvantage by Medicaid enrollment rates above state median, or<br>• participation in SNAP, WIC and other public benefits programs at rates above state median, or<br>• poor health status by life expectancy below the median for the state<br>• or percent of low birth weight above state median<br>• or an area determined to be "protected" by the Department AND areas that include schools, child care facilities, elder care facilities, community centers within 0.25 miles of nonmajor air quality permit applicant source OR Within 1 mile of a major air quality permit applicant source	This bill applies to "affected communities" defined as a U.S. census tract in which<br>• Median household income is ≤ 2 times the Federal poverty rate for a household of four; or<br>• The portion of the state's population that identifies as a race other than white, including individuals who identify their race or ethnicity as Hispanic or Latino, is greater than 35%;<br>AND<br>• A sources of proposed sources is located.
Consequences for Failure to Comply	None	None	None
Regulations	The Department may adopt regulations necessary to implement the subtitle.	The Department may adopt regulations to further define "protected communities"	Not addressed
Legislative Outcome	Senate Floor: passed 43- 3 on third reading with revisions.	House Environment and Transportation Committee: Unfavorable Report 20 – 0 (with 2	Senate Education, Health and Environmental Affairs Committee: Unfavorable Report 7 – 4.

	House Environmental Matters Committee: Unfavorable Report 15 – 0 (with 9 absent).	excused) and withdrawn by bill sponsor Delegate Lam.	
